# The role of lactate in cardiovascular diseases

**DOI:** 10.1186/s12964-023-01350-7

**Published:** 2023-11-03

**Authors:** Jun Ouyang, Hui Wang, Jiangnan Huang

**Affiliations:** 1 Department of Cardiology, The First Affiliated Hospital of Guangxi Medical University, Nanning, China; 2 School of Pharmacy, Guangxi Medical University, Nanning, China

**Keywords:** Lactate, Lactylation, Acute myocardial infarction, Heart failure, Atrial fibrillation, Atherosclerosis

## Abstract

Cardiovascular diseases pose a major threat worldwide. Common cardiovascular diseases include acute myocardial infarction (AMI), heart failure, atrial fibrillation (AF) and atherosclerosis. Glycolysis process often has changed during these cardiovascular diseases. Lactate, the end-product of glycolysis, has been overlooked in the past but has gradually been identified to play major biological functions in recent years. Similarly, the role of lactate in cardiovascular disease is gradually being recognized. Targeting lactate production, regulating lactate transport, and modulating circulating lactate levels may serve as potential strategies for the treatment of cardiovascular diseases in the future. The purpose of this review is to integrate relevant clinical and basic research on the role of lactate in the pathophysiological process of cardiovascular disease in recent years to clarify the important role of lactate in cardiovascular disease and to guide further studies exploring the role of lactate in cardiovascular and other diseases.

Video Abstract

**Supplementary Information:**

The online version contains supplementary material available at 10.1186/s12964-023-01350-7.

## Introduction

**Fig. 1:**
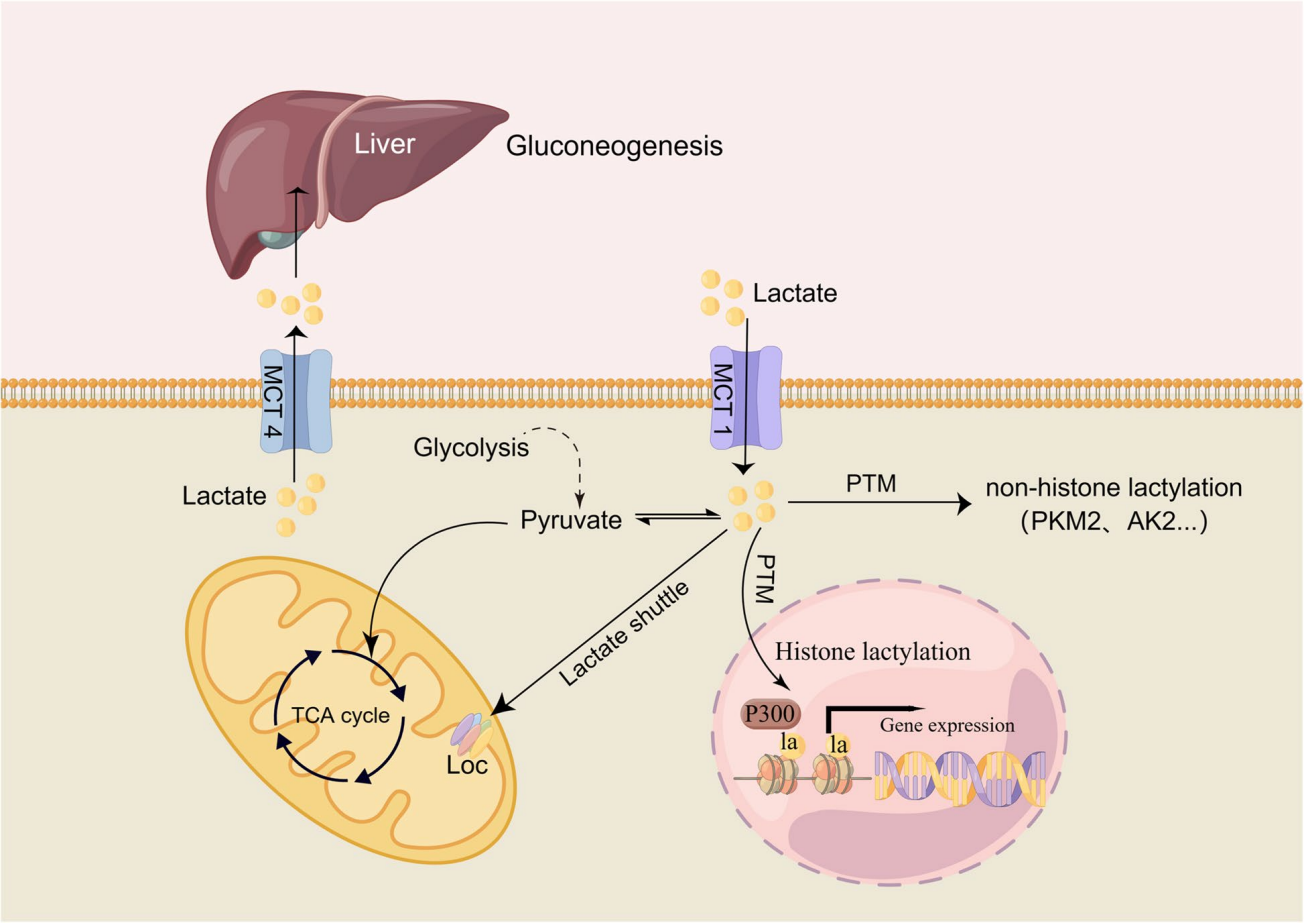
Schematic diagram of the lactate shuttle. In the cytoplasm, lactate is transformed into pyruvic acid under the catalysis of LDHB and then enters the mitochondrial tricarboxylic acid cycle (TAC). In addition, lactate can enter the mitochondria directly through the lactate oxidation complex (LOC) on the inner mitochondrial membrane and be converted into pyruvic acid. Lactate is also the precursor of gluconeogenesis. Lactate can be used as a substrate for posttranslational modification (PTM), causing the lactylation of histones and nonhistones, and can then regulate gene expression or protein function. Abbreviations: TAC: tricarboxylic acid cycle; PTM: posttranslational modification; LOC: lactate oxidation complex; PKM2: M2-type pyruvate kinase; AK2: adenylate kinase 2

Since the discovery of lactate in 1780, lactate has been considered a metabolic waste product generated from glycolysis that does not have any major physiological functions [1, 2]. With the deepening of research in recent years, the mysterious role of lactate has gradually been identified. In addition to representing a metabolic waste product, lactate is also involved in the growth and development of organs and in the coordination of vascular development and progenitor cell behaviour in the developing mouse neocortex [3]. It can also be used as the precursor of gluconeogenesis or can directly/indirectly enter the mitochondrial matrix to provide energy [4], act as a signalling molecule to maintain the homeostasis of subcellular organelles [5] and the crosstalk between neurons [6], modulate immunity through several signalling pathways [7–9], and even directly regulate protein function to control cell proliferation [10]. Even more surprising is the fact that lactate can serve as an epigenetic modification substrate, causing histone or nonhistone lysine residues to undergo lactylation, which then regulates gene transcription or protein function [11–14] (Fig. 1).

There are two sources of lactate in the body: one is mainly from glycolysis, which is catalysed by lactate dehydrogenase (LDHA/B), and the other is from glutamine catabolism [15]. There are three isomers of lactate, namely, D-lactate, L-lactate and racemic DL-lactate [16]. Among these, L-lactate is the most common form present in the body and is involved in several biological processes of the organism [16]. In recent years, it has also been found that D-lactate is also present in the body, mainly in the mitochondria [17]. The effect of D-lactate has been reported to be related to the transport of some metabolites in the body, such as H+, pyruvic acid and malate, due to the presence of D-lactate/H + cotransporter, D-lactate/pyruvic acid reverse transporter and D-lactate/malate reverse transporter in the mitochondria [18].

Monocarboxylate transporters (MCTs) are proteins that are responsible for lactate transport in the body. They are encoded by solute carrier family 16 (SLC16) genes. At present, 14 transmembrane proteins have been found, of which MCT1-4 are involved in lactate transport [9]. In addition to transport lactate, they can also transport pyruvate and ketone bodies (acetoacetate and β-hydroxybutyrate) [19–21]. Among these four transporters, MCT1 and MCT4 play pivotal roles in lactate transport [22]. MCT1 is responsible for the import of lactate, while MCT4 is responsible for its export, and they jointly maintain the intracellular balance in the lactate content and the pH value. Studies have confirmed that lactate signaling mediated by MCT1/4 participates in many biological processes. For example, MCT1 mediated lactate absorption can be involved in lipid metabolism and autophagy by activating the AMPK-SREBP1-SCD1 pathway [23]. On the other hand, research has found that autophagy can affect the transcription of MCT1 through the Wnt-β-catenin pathway [24]. In addition, MCT1 mediated lactate signaling can also participate in ferroptosis [23], angiogenesis [25], amino acid metabolism [26]. Research has found that MCT4 can also participate in autophagy [27]. And it can participate in cell cycle progression through the late apoptosis/necrosis pathway. Multiple factors can affect the expression of MCT4, such as oxidative stress, hypoxia inducible factor-1 (HIF-1) [28], and Butyrate [29]. However, both MCT2 and MCT3 have been poorly studied.

In addition to MCTs, another transporter family for lactate is the sodium-coupled monocarboxylate transporter family (SMCT) [9]. SMCT1 (SLC5A8) and SMCT2 (SLC5A12) are two SMCT family members that have been reported thus far, of which SMCT1 has been shown to transport lactate [30–34]. Additionally, GPR81, a member of the G protein-coupled receptor (GPR) family expressed on the cell membrane, has been shown to mediate lactate signalling [35–37]. Recent studies have reported that lactate transporters (MCTs and SMCTs) or receptors for lactate signal transduction (GPR81) are involved in the occurrence and development of a variety of diseases, especially cancers such as breast cancer, lung cancer, bowel cancer, bladder cancer, ovarian cancer, prostate cancer, and glioma [38–43].

Clinical and basic studies have found that in many cardiovascular diseases, such as myocardial infarction, heart failure, atrial fibrillation (AF) and atherosclerosis, the level of lactate and the function or expression of MCTs are altered. Artificial supplementation or a reduction in the body’s lactate content or manipulation of MCTs at the organismal or cellular level affects intracellular and extracellular lactate levels and significantly affects the disease status. Many clinical studies have also shown that lactate is a very important prognostic indicator of cardiovascular diseases [44–46]. Therefore, lactate also plays a significant role in the field of cardiovascular diseases. A detailed and comprehensive understanding of the specific role of lactate and its transporters in cardiovascular diseases could reveal a novel strategy for clinicians to treat cardiovascular diseases in the future. In this article, we summarize the role of lactate in myocardial homeostasis and also its involvement in myocardial infarction, heart failure, AF and atherosclerosis.

## Lactate in myocardial homeostasis

Lactate is an important energy source in organs such as the heart, brain, and muscles [47–49]. For example, for the heart, even under normal circumstances, lactate oxidation accounts for over 50% of the total cellular energy supply [50].Under normal physiological conditions, cardiomyocytes mainly rely on fatty acid oxidation for energy supply, accounting for approximately 60–90% of total energy [51]. Glucose provides approximately 10–40% of energy [52]. cardiomyocytes, like “omnivores”, are also powered by lactate, amino acids, and ketone bodies [51]. Among these substances, cardiomyocytes prefer to use lactate for energy supply, which can provide approximately 10% of the energy [53]. Especially during exercise, lactate can provide over 50% of the energy required by cardiomyocytes [54]. During exercise, lactate production increases. To promote the uptake of more lactate by cardiomyocytes and provide energy to cardiomyocytes, lactate also can serve as an important physiological signalling molecule, affecting the expression of MCT1 protein and mitochondrial biogenesis by activating a series of transcription networks [55]. However, for 24-month-old cardiomyocytes, the decrease in LDH enzyme activity leads to a decrease in lactate oxidative energy supply, which in turn leads to an increase in compensatory fatty acid oxidative energy supply. It is the above phenomenon, named metabolic remodelling, that leads to a decrease in the function of myocardial cells [56].

For cardiomyocytes, lactate is definitely not just as a simple end waste product of glycolysis metabolism. In addition to being an important energy source for cardiomyocytes, lactate also plays other significant physiological functions in maintaining cardiomyocytes homeostasis. Research has found that lactate can promote the maturation and regeneration of mouse neonatal cardiomyocytes and human pluripotent stem cell (hPSC)-derived cardiomyocytes by upregulating the expression of *BMP10*, *LIN28*, and *TCIM* while downregulating the expression of *GRIK1* or *DGKK* and different ion channels [57]. The authors speculate that the mechanism of action of lactate may be achieved by directly influencing epigenetic modifications such as acetylation and lactylation modification. Previous studies have also reported that lactate and pyruvate can protect cardiomyocytes from oxidative stress by scavenging free radical species [58, 59]. In addition, research has found that lactate can affect the electrophysiological activity of cardiomyocytes. For example, lactate can activate ATP-sensitive potassium ion channels in guinea pig ventricular myocytes and shorten the action potential duration [60]. The possible mechanisms by which lactate activates potassium channels include the following: (1) lactate can interfere with ATP production by inhibiting glycolysis; (2) lactate, like ADP, can reduce the sensitivity of ATP-sensitive potassium ion channels to ATP [61, 62]; (3) lactate can serve as an ion channel opener, similar to cromakalin [63] and pinacidil [64]; and (4) specific cell components are needed. However, in a recent study, it was found that lactate also can prolong the repolarization of action potentials by altering the redox state of nucleotides in cells [65].

In addition to cardiomyocytes in the heart, another type of cell in the heart, namely, cardiac fibroblasts, is often considered a negligible component, mainly because cardiac fibroblasts make up less than 25% of the total mass of the heart [66]. However, these cells are not negligible, as cardiac fibroblasts can maintain the homeostasis of cardiac structure and function by secreting extracellular matrix (ECM) [67]. Furthermore, the metabolic coupling formed by the lactate shuttling between cardiac fibroblasts and cardiomyocytes is crucial for maintaining the overall homeostasis of the heart. Lactate shuttling between fibroblasts and cardiomyocytes improves the metabolism of cardiomyocytes by changing the expression and subcellular localization of myocardial cell metabolism-related proteins (HK-2, PKM2, LDHA/B, HIF-1a, GLUT-1, FBP2, TIGAR and PGAM2) [68].

In summary, lactate is important for maintaining myocardial homeostasis and when studying cardiac metabolism and seeking treatment for heart diseases, cardiac fibroblasts need to be given the same importance as cardiomyocytes.

## Heart failure

In recent years, basic and clinical studies have reported the role of lactate in heart failure. Many studies have shown that high levels of lactate in the blood are a marker of poor prognosis in patients with heart failure [44, 45]. The role of lactate in acute and chronic heart failure seems to be different. Blood lactate levels significantly increase during acute heart failure, while in patients with chronic heart failure, there is little change in their blood lactate levels [69]. High levels of lactate at admission are associated with a high mortality rate in patients with acute heart failure (AHF) [45]. The main reasons for the increase in blood lactate levels in heart failure patients include a decrease in blood oxygen being transported to peripheral tissues or a decrease in the tissue’s ability to absorb oxygen [70], activation of the neurohumoural system (the adrenal and sympathetic nervous systems) [71, 72], an increase in the demand for oxygen consumption and organ dysfunction (liver or kidney dysfunction) that dysregulates lactate clearance [45, 70, 73] and diaphragmatic fatigue [74]. Many studies have shown that lactate accumulation in patients with acute heart failure is not caused by respiratory dysfunction or hypoxemia but is related to the heart index. The strongest determinants of lactate accumulation are mixed venous oxygen saturation, heart rate and systemic vascular resistance [70].

Perhaps during acute heart failure, the increase in lactate levels is a self-protection mechanism of the body towards the cardiovascular system. Similar to congestive heart failure, angiotensin-converting enzyme inhibitors are known to alter cardiomyocytes from a lactate-producing state to a lactate-consuming state, thereby improving myocardial metabolism and providing energy to cardiomyocytes. The intracellular lactate shuttle may be involved in the process of providing energy for cardiomyocytes [4]; that is, lactate may shuttle into cardiomyocytes mitochondrial matrix through the lactate oxidation complex (LOC) consisting of MCT, BSG or CD147, LDH and cytochrome oxidase (COX) [16] on the inner mitochondrial membrane, be converted into pyruvic acid, and then enter the tricarboxylic acid cycle (TAC) to generate ATP (Fig. 1). In addition, lactate can also be directly transformed into pyruvic acid in the cytoplasm or to glycogen through gluconeogenesis (Fig. 1). Previous studies have shown that monocarboxylic acid transporter 1 (MCT1), which is responsible for lactate absorption, is upregulated in cardiomyocytes of rats with congestive heart failure, thereby increasing the absorption of lactate by cardiomyocytes [50]. In addition, inhibiting monocarboxylic acid transporter 4 (MCT4), the transporter responsible for lactate excretion by cardiomyocytes, has been shown to improve the hypertrophy of mouse cardiomyocytes by restoring pyruvic acid flux and improving oxidative stress in the cytoplasm [75].

Cardiac fibroblasts also play a crucial role in heart failure. Metabolic remodelling is a key pathological mechanism by which cardiac fibroblasts are activated and transformed into myofibroblasts, leading to cardiac fibrosis [76]. Metabolic remodelling of cardiac fibroblasts refers to a shift from fatty acid oxidative phosphorylation to glycolysis. Lactate, the end product of glycolysis, is essential for the activity of proline hydroxylase, TGF-β1 and the hydroxylation of collagen, which is an important molecule for the activation of cardiac fibroblasts [77]. In the heart failure process, the study has found that lactate can regulate the production of inflammatory cytokines by cardiac fibroblasts, reducing the production of Fas, Fraktalkine, or IL-12p40 and stimulating IL-13 and SDF1a [57].

Lactate shuttling between cardiac fibroblasts and cardiomyocytes is important for maintaining myocardial homeostasis which was mentioned above, but this phenomenon is disrupted in mice with age-related heart failure [68]. Additionally, Lactate shuttling between cardiomyocytes and cardiac fibroblasts also occurs in the process of hypertensive heart remodelling, the study has found that the mitochondria-rich GCN5-like 1 (GCN5L1) protein causes acetylation of mitochondrial pyruvate carriers (MPCs), leading to a decrease in fatty acid oxidative phosphorylation in cardiac fibroblasts and thereby promoting increased glycolysis and activation of cardiac fibroblasts. At the same time, lactate produced by cardiac fibroblasts enters the cells through MCT1 on the surface of cardiomyocytes, leading to cardiomyocytes hypertrophy [78].

Recently, Yingxian Sun’s team found that a reduction in α-MHC-k1897 lactylation modified by lactate can cause heart failure [79]. This further demonstrates the importance of lactate for myocardial homeostasis in heart failure. Therefore, clearing lactate during acute heart failure is not a wise strategy; however, some studies have also shown that lactate causes harm to the myocardium [80]. Indeed, a previous study reported that infusion of half-molar sodium lactate to AHF patients improved their cardiac output [81]. In conclusion, lactate is not just a simple end product of glycolysis but plays several important physiological functions during AHF, as mentioned earlier.

## Myocardial infarction

Acute myocardial infarction (AMI) is one of the most serious coronary artery diseases caused by myocardial ischaemia and necrosis due to coronary artery stenosis and occlusion [82, 83]. Coronary occlusion leads to a sharp decrease in blood oxygen that is transported to myocardial cells, thus leading to a decrease in mitochondrial oxidative phosphorylation and an increase in the glycolytic rate of myocardial cells [46]. Naturally, this leads to an increase in the production of lactate in cardiac myocytes, which is consistent with many clinical studies that have found an increase in circulating lactate levels in patients with myocardial infarction [46, 84]. Many clinical studies have confirmed that circulating lactate levels have great prognostic value for predicting adverse clinical outcomes in patients with myocardial infarction [46, 84–86]. As early as 1991, Zarko Mavri C et al. found that peripheral blood lactate levels could predict the occurrence of shock in patients with AMI [84]. In addition, in patients with ST myocardial infarction, a high lactate concentration (> 1.8 mmol/l) at admission has been associated with 30-day mortality and poor response to percutaneous coronary intervention (PCI) [46]. When the blood lactate concentration is > 4.0 mmol/l at admission, the mortality rate sharply increases [46]. Additionally, another study reported that in ST patients with myocardial infarction who already received PCI treatment, blood lactate could only predict early mortality in patients with severe haemodynamic disorders [87]. Furthermore, preoperative lactate (> 4.0 mmol/l) can also predict early and late mortality after AMI with cardiogenic shock and acute coronary artery bypass grafting [85]. However, a study has shown that lactate (≥ 2.5 mmol/l) can also be used as a prognostic factor in patients with AMI complicated with mild to moderate heart failure but without obvious haemodynamic changes and a lack of hypotension signs [86]. Compared to baseline lactate levels, 24-hour lactate levels and 24-hour lactate clearance rates have a better ability to predict hospitalization-associated mortality in patients with AMI [88]. This study showed an increase in the 24-hour lactate clearance rate, which was associated with a decrease in hospitalization mortality in patients with AMI [88]. Due to various factors that can affect blood lactate levels in patients with myocardial infarction, the factors underlying the increase in blood lactate levels in patients with AMI are very complex to identify. Potential causes for elevated blood lactate levels in patients with AMI include increased glycolysis, tissue hypoperfusion, insulin resistance, haemodynamic impairment, myocardial infarction, blood glucose values, reduced ability of the body to remove lactate, uncontrolled diabetes stimulating the production of fatty tissue [89] and renal lactate [90], the secretion of stress hormones during AMI, circulatory failure, and metformin treatment [73]. The above factors may be intertwined to promote an increase in lactate levels in patients with AMI.

Despite the above findings, there are currently few basic research studies exploring the relationship between lactate and myocardial infarction. Under physiological conditions, lactate upregulates the expression of lactate oxidation complex (LOC)-related genes by increasing the intracellular expression of reactive oxygen species (ROS) and nuclear factor erythroid 2-related factor 2 (NRF-2) [91, 92]. In addition, lactate relaxes the coronary arteries in a nitric oxide (NO)-dependent manner to regulate cardiac blood flow [93]. However, a study has shown that the infusion of lactate into male Wistar rats with chronic myocardial infarction does not improve cardiac function [94]. In contrast, lactate inhibits the activity of antioxidant enzymes in the heart and inhibits the expression of LOC-related genes, such as MCT1 [94]. Furthermore, lactate, through lactylation, also participates in the development of AMI. It has been reported that in the early stage of AMI, lactylation of H3K18la in monocytes induces the expression of cardiac repair genes such as Lrg1, Vegf-a and IL-10, thereby improving the repair of infarcted hearts [95]. However, after AMI, the lactylation of the transcription factor Snail1 in endothelial cells promotes TGF-β gene expression and leads to endothelial-to-mesenchymal transition (EndoMT), causing detrimental effects on cardiac repair [96]. Taken together, there is controversy over the beneficial role of lactate in patients with AMI. The precise effect of lactate may be dependent on the cell types that are involved in myocardial infarction, the disease stage, the presence of concomitant diseases, etc.

## Atrial fibrillation

Atrial fibrillation (AF) is the most common type of arrhythmia and is characterized by rapid and irregular activation of atrial myocytes, leading to a series of cardiovascular diseases, such as heart failure and stroke, with high mortality rates [97, 98].

Research has found that atrial remodelling may be the structural basis leading to the maintenance and recurrence of AF [99, 100]. Atrial remodelling includes three aspects: electrical/contractile remodelling, metabolic remodelling, and structural remodelling [100]. Electrical remodelling refers to the shortening of the refractory period during AF, which helps to increase the stability of atrial fibrillation [100]. Contractile remodelling refers to the decrease in atrial contractile capacity leading to a decrease in atrial contractile function after AF cardioversion [100]. Research has found that the two types of remodelling mentioned above go hand in hand, resulting from a common mechanism of the reduction in L-type Ca^2+^ currents [100, 101]. Metabolic remodelling refers to the change in the substances used by atrial myocytes for oxidative energy supply, that is, a shift from using fatty acids for energy supply to using glucose for energy supply. Structural remodelling refers to the hibernation phenotype of atrial myocytes, which enter a dedifferentiated state, mainly manifested as increased cell volume and the accumulation of perinuclear glycogen [102–104]. The above three remodelling mechanisms lead to the formation of AF substrates.

Previous studies have found that lactate signalling cascades that are significantly associated with cardiovascular diseases such as ischaemic-perfusion injury and heart failure are also involved in the process of atrial structural remodelling during AF [50, 99, 105, 106]. During AF, especially persistent atrial fibrillation (PeAF), there is a significant increase in lactate in the right atrial appendage tissue [99]. At the same time, the expression of MCT1 on the mitochondrial membrane is also significantly increased. The additional lactate enters the mitochondria through MCT1 on the mitochondrial membrane, causing mitochondrial oxidative stress and triggering mitochondrial control of apoptosis, such as the increase in the expression of cytochrome oxidase C and cleaved caspase 3 and cleaved caspase 9 [99]. This response implies that lactate is not only a manifestation of insufficient tissue oxygen supply but also has its own unique biological role, affecting the occurrence and development of diseases. Metabolomics and proteomics analysis showed that the glycolysis pathway in the enrichment pathway is associated with valvular atrial fibrillation, and DL-lactate plays a crucial role in valvular atrial fibrillation [107]. Taken together, targeted glycolysis or lactate production and transport may be used for the treatment of AF. It is currently unclear whether lactylation also participates in the occurrence and development of AF.

## Atherosclerosis

**Fig. 2:**
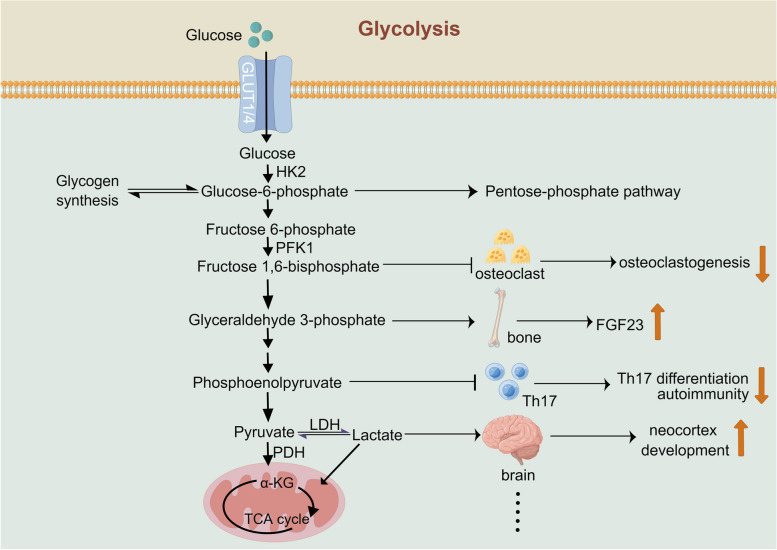
Schematic diagram of the physiological function of metabolites in the glycolysis pathway. A variety of metabolites produced during glycolysis have very important physiological functions. For example, glucose-6-phosphate is the precursor of gluconeogenesis, and it can enter the pentose phosphate pathway; fructose 1,6-bisphosphate can inhibit osteoclastogenesis; glyceraldehyde phosphate can promote the production of FGF23 by bone; phosphoenolpyruvate can inhibit the differentiation of TH17 cells and regulate the autoimmune system; and lactate can promote the development of the cerebral neocortex. Abbreviations: FGF23: fibroblast growth factor 23; HK2: hexokinase; PFK1: phosphofructokinase-1; PDH: pyruvate dehydrogenase; TAC: tricarboxylic acid cycle; LDH: lactate dehydrogenase

Atherosclerosis is a chronic inflammatory process involving a variety of cells, including vascular endothelial cells, vascular smooth muscle cells (VSMCs), monocytes, macrophages and lymphocytes. Studies have shown that glycolysis plays a very important role in the development of atherosclerosis, and aerobic glycolysis seems to play a more important role than anaerobic glycolysis [108]. Vascular endothelial cells and inflammatory immune cells, such as macrophages and VSMCs, are more prone to undergo aerobic glycolysis even under normoxic conditions [109–115]. Aerobic glycolysis provides more ATP than mitochondrial oxidative phosphorylation in the same period [116]. In addition, different metabolites in the glycolytic pathway also participate in regulating various processes in the body (Fig. 2) [3, 117–119]. In the past few years, studies have implicated lactate, the end product of glycolysis, in playing a vital and complex role in the development of atherosclerosis. A cross-sectional study involving 1496 participants reported that blood lactate levels were positively associated with carotid atherosclerosis, independent of other cardiovascular risk factors [120]. Thus, lactate must be related to atherosclerosis, similar to myocardial infarction, heart failure and AF. As mentioned above, VSMCs, vascular endothelial cells and macrophages are mainly involved in the occurrence and development of atherosclerosis, so how lactate acts on these three types of cells and thus affects the occurrence and development of atherosclerosis is the topic of our next discussion.

### Lactate in vascular smooth muscle cells

Many basic studies have shown that lactate acts on a variety of cells, such as vascular endothelial cells, VSMCs, macrophages and lymphocytes, during atherosclerosis through a variety of complex intracellular pathways. For example, studies have shown that downregulation of MCT3 (responsible for the reabsorption of lactate) expression caused by DNA methylation in VSMCs during atherosclerosis leads to the obstruction of lactate transport [121]. This process has been shown to promote the transformation of VSMCs from a contraction phenotype to a proliferative/secretory phenotype [121]. The demethylating agent 5-aza-2-deoxycytidine has been shown to restore the expression of MCT3 and lactate transport, as well as to improve the phenotype of VSMCs [121]. This study indicates that lactate is beneficial to the phenotype of VSMCs. However, in another study, inhibiting LDHA- or PKM2-dependent glycolysis in VSMCs was shown to improve their proliferation and migration phenotype, thereby inhibiting atherosclerosis [122, 123]. In addition, κ-opioid receptor (κ-OR) agonists such as U50488H have been shown to reduce the osteogenic differentiation and calcification of VSMCs by inhibiting intracellular 6-phosphofructo-2-kinase/fructose-2,6-bisphosphatase 3 (PFKFB3), a key enzyme in the processes regulating glycolysis and the lactate content [124]. Furthermore, lactate also accelerates mitochondrial fission through the NR4A1/DNA-PKcs/p53 signalling pathway and inhibits mitochondrial autophagy, leading to osteogenic transformation and the calcification of VSMCs [125]. Mitochondrial damage has been shown to occur during atherosclerosis. Thus, lactate plays a multifaceted role in VSMCs. During atherosclerosis, blood lactate levels in the body are elevated; moreover, the glycolytic rate of VSMCs is also higher [108, 120]. However, it is unclear whether the lactylation mentioned above also occurs in VSMCs. Targeting lactylation may serve as a potential therapeutic strategy to treat atherosclerosis in the future.

### Lactate in endothelial cells

Under physiological conditions, endothelial cells are in a resting state compared to other metabolically active cells. Due to their low energy demand, endothelial cells contain a relatively lower number of mitochondria [114]. Therefore, for endothelial cells, most of the energy requirements are met by glycolysis [115]. Theoretically, lactate levels in endothelial cells are generally higher than those in other cells due to their high glycolysis influx. As previously discussed in the context of vascular endothelial cells, lactate also plays a complex and multifaceted role in endothelial cells during atherosclerosis. Studies have reported that lactate enters endothelial cells through MCT1, activates NF-KB and HIF-1α, and regulates the transcription of genes such as IL-8 and vascular endothelial growth factor (VEGF) [126]. In addition, lactate also promotes the phosphorylation of pyruvate kinase receptor (PKR) and activates the inflammatory process, thus aggravating the occurrence of atherosclerosis [127]. However, lactate also induces the expression of the key atherosclerosis protective transcription factor Krüppel-like factor 2 (KLF2) gene by acting on G protein-coupled receptor 81 (GPR81), a lactate sensor [128]. This action plays a protective role in endothelial cell inflammation caused by oscillatory shear stress (OSS) in the vascular injury region of atherosclerosis [128]. Furthermore, recent studies have reported that lactate can also be used as an epigenetic modifying substrate to cause lactylation in the process of atherosclerosis, thus affecting the occurrence and development of atherosclerosis. Exercise increases lactate levels in the body, and this increased lactate enters endothelial cells through the MCT transporter, causing Mecp2 lysine lactylation (Mecp2k271la). Mecp2 lysine lactylation then inhibits the production of inflammatory mediators through the Ereg/MAPK signalling pathway, thus inhibiting the occurrence of atherosclerosis [13].

### Lactate in macrophages

Macrophages are the main type of inflammatory immune cells involved in atherosclerosis [129]. At present, studies exploring the role of lactate in macrophages during atherosclerosis are lacking. However, the role of lactate in macrophages has been extensively explored in the field of oncology. Most studies have shown that lactate mainly regulates the polarization of macrophages [130–132]. Depending on the different environmental stimuli, macrophages mainly exist in either the M1 or M2 state [133]. M1-type macrophages are mainly proinflammatory, while M2 macrophages are mainly anti-inflammatory and are related to tissue damage repair and healing. At present, the vast majority of studies have shown that lactate mainly promotes the transformation of macrophages into the M2 phenotype through various cellular signal transduction pathways. For example, lactate inhibits the Toll-like receptor 4 (TLR4)-dependent macrophage proinflammatory response that is induced by LPS [134]. This protective effect may be mediated by G protein-coupled receptor 81 (GPR81) and inhibition of YAP and NF-kB [134]. In addition, studies have shown that lactate also activates the macrophage M2-like phenotype through G protein-coupled receptor 132 (GPR132) [135]. The potential cellular signalling pathways involved in the lactate-mediated transformation of macrophages to the M2-like phenotype include the cAMP/CREM, mTORC1/ATP6V0D2/HIF-2α, Nrf2, PI3K/AKT, and ERK/STAT3 pathways [136, 137]. These signal transduction pathways may be activated by GPR81, GPR132, Olfr78 and MCTs expressed on the surfaces of macrophages [131, 134, 135, 138, 139]. Recent studies have shown that epigenetic modification, also known as lactylation, is also involved in the M2-like phenotype of macrophages induced by lactate [11, 140]. Under LPS stimulation, the glycolytic rate of macrophages increases, leading to an increase in lactate levels. Subsequently, lactate generates lactyl-CoA, and histone lysine residues are lactylated under the action of the writer protein (P300) [11]. It is believed that during atherosclerosis, the glycolytic rate of macrophages in the plaque is also elevated. However, it remains unclear whether lactylation also participates in the polarization of macrophages, thus affecting the onset and development of atherosclerosis.

**Fig. 3:**
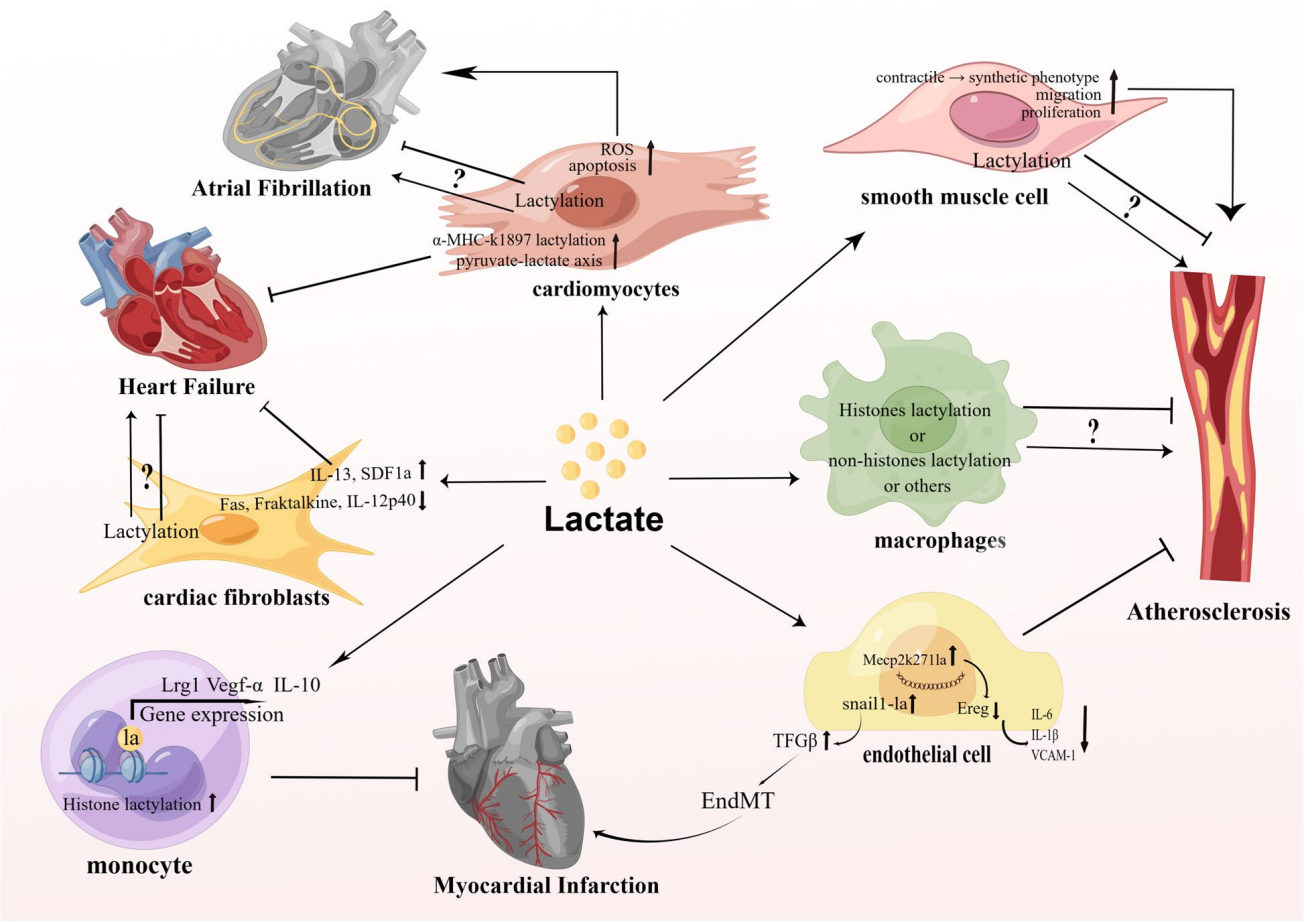
Schematic diagram of the role of lactate in heart failure, myocardial infarction, atrial fibrillation and atherosclerosis. In heart failure, the increase in lactate levels in myocardial cells can improve myocardial metabolism through the pyruvate-lactate axis, thereby improving myocardial hypertrophy. In addition, recent studies have found that α-MHC-K1897la lactylation can improve sarcomeric structure and function, thereby alleviating the development of heart failure. Furthermore, lactate can regulate the production of inflammatory cytokines by myocardial fibroblasts, reducing the production of Fas, Fraktalkine, or IL-12p40 and stimulating IL-13 and SDF1a to improve heart failure. It is unclear whether lactylation in myocardial fibroblasts involve in heart failure. In myocardial infarction, the early increase in the level of H3K18la in monocytes can promote the expression of repair genes (Lrg1, Vegf-α, and IL-10), thereby improving myocardial infarction. In addition, in the later stage, the increase in snail1 lactylation in vascular endothelial cells can promote the expression of tfg genes, thereby promoting the occurrence of EndMT and exacerbating the occurrence of myocardial infarction. In atrial fibrillation, high level of lactate can stimulate myocardia cells to produce ROS and cause myocardia cells apoptosis. It is unclear whether lactylation in myocardial fibroblasts involve in atrial fibrillation. In atherosclerosis, lactate can promote the proliferation and migration of smooth muscle cells and phenotypic transformation, thus promoting the occurrence of atherosclerosis. The increased level of Mecp2-k271la in vascular endothelial cells can inhibit the expression of the Ereg gene and the production of inflammatory mediators (IL-6, IL-1β, and VCAM-1), thereby inhibiting the occurrence of atherosclerosis. At present, it is not clear whether lactate can affect the occurrence of atherosclerosis by affecting histone or nonhistone lactylation or by other unknown mechanisms in macrophages. Abbreviations: EndMT: endothelial-to-mesenchymal transition

A brief graphic abstract of the role of lactate in cardiovascular diseases such as heart failure, AMI, AF and atherosclerosis is shown in Fig. 3.

## Lactylation

Posttranslational modifications (PTMs) are very important epigenetic phenomena in the body that can alter the structure, activity, stability, and cellular localization of proteins and participate in various physiological and pathological processes. Common PTMs include acetylation, phosphorylation, ubiquitination, methylation, and glycosylation.

In addition to the abovementioned PTMs, emerging studies have found that metabolites are closely linked to PTMs [141, 142]. Currently, research has found that PTMs related to metabolites include propionylation and butylation [143], succinylation [144],crotonylation [145],2-hydroxyisobutylation [146], and benzoylation [147], as well as formylation [148],malonylation [149], and glutarylation [150]. Moreover, in 2019, Zhao et al. found through high-performance liquid chromatography-tandem mass spectrometry analysis that lactate, the end product of glycolysis, can provide L-lactyl that can be added to the lysine residue of histones, named lactylation modification [11]. Under LPS stimulation, the level of H3k18la in macrophages was enhanced, driving the expression of the *Arg1* and *Vegfa* genes. When LDHA or glycolysis is inhibited, the level of histone lactylation in macrophages is significantly reduced, followed by a decrease in the expression of *Arg1* and *Vegfa* [11]. This further proves the existence of cross talk between the metabolome and epigenetics. In addition, shortly after the discovery of histone lactylation, nonhistone lactylation was discovered, showing the universality of lactylation [142]. Overall, lactylation may affect protein function through two pathways: (1) histone lactylation can directly bind to the promoter region of a specific gene, thereby affecting gene expression; and (2) lactylation directly modifies proteins, which can regulate their biological activity.

Interestingly, according to recent research, lactylation, similar to acetylation, is modulated by the same “writer proteins”, such as P300/CBP, and “eraser proteins”, such as HDAC1-3 [11, 151]. This finding may indicate that lactylation and acetylation are coordinated in a temporal and spatial fashion to affect cellular biological processes. Which specific type of modification dominates may depend on the proportion of substrates (acetyl-CoA and lactyl-CoA) [152], the recruitment of different cofactors, changes in modification kinetics [153], or the activity of specific modification enzymes. Unfortunately, it has not been determined whether there are specific lactylases or delactylases.

In recent years, studies have found that histone or nonhistone lactylation is not only involved in the occurrence of cardiovascular diseases, such as myocardial infarction, heart failure and atherosclerosis (we have discussed this research in the corresponding sections), but is also involved in the occurrence and development of other diseases, such as tumours (ocular melanoma [154], clear cell renal cell carcinoma [155], prostate cancer [156], colorectal cancer [157], hepatocellular carcinoma [158], and breast cancer [159]), infections (sepsis) [140, 160], nervous system diseases (Alzheimer’s disease [161] and ischaemic stroke [162]), kidney diseases (acute kidney injury [AKI] [163]), liver diseases (liver ischaemia‒reperfusion [LI/R] injury [12], liver fibrosis [164] and nonalcoholic fatty liver disease [165]), lung diseases (lung fibrosis [166] and pulmonary hypertension [167]), metabolic diseases (insulin resistance [168]) and retinal neovascular diseases [169]. In addition, lactylation is also involved in normal physiological processes, such as autophagy [14, 170], osteoblast differentiation [171], and growth and development processes, such as neuronal [172] and embryonic development [173, 174]. Overall, lactylation plays a crucial role in the field of cardiovascular disease and even other diseases.

Nevertheless, research on lactylation is still in its infancy. In the future, we need to explore the specific enzymes involved in lactylation and to clarify the coordination between lactylation and other PTMs. Furthermore, whether only lysine can undergo lactylation still requires us to address this issue. This will help us develop strategies for better regulating lactylation to treat cardiovascular and other diseases in the future.

## Drug targets and applications

Several drugs that inhibit the production of lactate are currently available. They include drugs that inhibit LDHA (responsible for the conversion of pyruvic acid to lactate), such as FX-11, GSK2837808A and vitamin C, or dual inhibitors of LDH, such as stiripentol, galloflflavin, N-hydroxyindoles, and AT-101 (gossypol) [16, 175]. Of course, there are also drugs that inhibit glycolysis (2-DG and lonidamine targeting hexokinase [HK]) or promote the entry of pyruvic acid into the citric acid cycle (dichloroacetate [DCA] inhibiting pyruvate dehydrogenase kinase [PDK]) to indirectly reduce the production of lactate [16]. Furthermore, it was recently demonstrated that lonidamine also inhibits MCT1/2/4 [176, 177]. Moreover, lactate transporter inhibitors such as AZD-3965, α-cyano-4-hydroxycinnamate, the MCT1/2 inhibitor AR-C155858 and the MCT1/4 inhibitors syrosingopine and meplazumab can also be used to alter lactate levels [16]. In addition to the above inhibitors, phloretin thalidomide and its derivatives (lenalidomide and pomalidomide) are known to inhibit MCT1 [178]. The above drugs have mostly been studied in the field of oncology and have been shown to exert anticancer effects. Currently, there are few drugs targeting SMCT. Some commonly used drugs in clinical practice seem to have MCT/SMCT inhibitory effects, such as the nonsteroidal anti-inflammatory drugs (NSAIDs) ibuprofen and salicylic acid [179, 180]. It is encouraging that some of the aforementioned drugs are already in the clinical trial stage, such as AZD-3965, meplazumab, AT-101 (gossypol), 2-DG, DCA and lonidamine. Stiripentol has been approved by the Food and Drug Administration (FDA) for treating epilepsy and Dravet syndrome [181, 182]. Therefore, drugs targeting lactate transporters may offer novel therapeutic strategies for the treatment of cardiovascular diseases.

Lactylation was first proposed by Zhao Yingming’s team, and this study was published in the journal “Nature” in 2019 [11]. We now know that lactylation is regulated by the histone acetyltransferase and histone deacetyltransferase families of proteins. The histone acetyltransferase family includes P300, CBP, GCN5, PCAF, MOF and TIP60 for lactylation [11, 14], while the histone deacetyltransferase family includes HDAC1-3 and SIRT1-3 for delactylation [151]. Therefore, lactylation can be regulated by manipulating these two enzyme families. Drugs that target histone acetyltransferases include the small molecule C646, the natural product curcumin, MB298 and A-485, which mainly inhibit P300 and CBP proteins, while trichostatin A (TSA), sodium butyrate and apicidin are known to inhibit HDAC1-3, and nicotinamide (NAM) inhibits SIRT1-3. At present, studies have shown that the level of lactylation increases in patients with AMI, heart failure and atherosclerosis, which may affect disease progression. Therefore, regulating lactylation may provide a promising strategy to treat myocardial infarction and heart failure and atherosclerosis in the future.

## Summary

Targeting the production or transport of lactate, manipulating epigenetic modification induced by lactate or altering lactate levels in the body may offer a new strategy for the treatment of cardiovascular diseases in the future. Notably, the modulation of lactylation modification seems to have great prospects in the treatment of cardiovascular diseases and other diseases in the future. Of course, as mentioned above, close attention must be paid to the disease stage and the types of cells involved in the treatment process. Further basic and clinical studies are required to validate the role of lactate in cardiovascular diseases and to confirm its therapeutic potential for treating patients.

## Data Availability

Not applicable.

## Abbreviations

AF: Atrial fibrillation
AHF: Acute heart failure
AK2: Adenylate kinase 2
AMI: Acute myocardial infarction
COX: Cytochrome oxidase
EndoMT: Endothelial-to-mesenchymal transition
FGF23: Fibroblast growth factor 23
GPR: G protein-coupled receptor
HK2: Hexokinase
LDH: Lactate dehydrogenase
LOC: Lactate oxidation complex
MCTs: Monocarboxylate transporters
NO: Nitric oxide
OSS: Oscillatory shear stress
PDH: Pyruvate dehydrogenase
PFK1: Phosphofructokinase-1
PKM2: M2-type pyruvate kinase
PKR: Pyruvate kinase receptor
PTM: Posttranslational modification
ROS: Reactive oxygen species
SLC: Solute carrier
SMCT: Sodium-coupled monocarboxylate transporter
TCA: Tricarboxylic acid cycle
TLR: Toll-like receptor
